# Association between timed up and go test and future incidence of disability: A nationwide representative longitudinal study in Korea

**DOI:** 10.1371/journal.pone.0270808

**Published:** 2022-07-05

**Authors:** Ki Young Son, Dong Wook Shin, Ji Eun Lee, Sang Hyuck Kim, Jae Moon Yun, Belong Cho

**Affiliations:** 1 Department of Family Medicine, Asan Medical Center, Seoul, Korea; 2 Department of Family Medicine/Supportive care Center, Samsung Medical Center, Sungkyunkwan University School of Medicine, Seoul, South Korea; 3 Center for Clinical Epidemiology, SAIHST, Sungkyunkwan University, Seoul, Korea; 4 Department of Family Medicine, Seoul National University Hospital, Seoul, Korea; 5 Health Promotion Center, Seoul National University Hospital, Seoul, Korea; 6 Department of Family Medicine, Bumin Hospital, Seoul, Korea; 7 Institute on Aging, Seoul National University College of Medicine, Seoul, Korea; University of Alberta, CANADA

## Abstract

Although previous studies examined the association between mobility and disability, they have used either subjective measure disability such as activity of daily living or instrumental activity of daily living or indirect measure such as long-term care service use with small size of participants. This study aimed to examine the association between timed up and go (TUG) test and disability incidence with national disability registration data in Korea longitudinally, by using a national representative sample. We used the National Health Insurance Service–National Health Screening Cohort (NHIS–HEALS) database of National Health Information Database. The NHIS–HEALS dataset includes disability information of National Screening Programme participants, including registration date and type of disability, which is merged from Korean National Disability Registry (KNDR). We used Cox proportional hazard models to evaluate the association between TUG and disability incidence. We constructed three models with different levels of adjustment; Model 3 was a fully adjusted model. We conducted subgroup analysis according to the risk factors for disability. The study population comprised 81,473 participants; 86 of them were newly registered to KNDR, which were observed during a mean follow-up of 4.1 ± 2.6 (maximum, 8.9) years. For 334,200.9 person-year (PY) follow-up, the disability incidence rate was 0.208 per 1,000 PY. Disability incidence was significantly higher in participants with abnormal TUG results than in those with normal TUG results. (adjusted hazard ratio [aHR] 1.600, 95% confidence interval [CI] 1.036–2.472). In subgroup analysis, the disability incidence increased in participants of normal cognition, without obesity or without cardiovascular (CV) disease. Increased incidence in disability was noted in participants with abnormal TUG results. The increase was more evident for participants with normal cognition, without obesity or CV disease.

## Introduction

According to Korean National Disability Registry (KNDR), over 2.4 million people were registered as people with disability, which comprises nearly 5% of Korean population [1]. These people are vulnerable population who need special attention for health care. Previous studies reported that people with disability often have adverse health behaviours [2–7], received fewer preventive health care services [8–12] and had more chronic diseases [2, 13–16]. Consequently, total mortality and cardiovascular (CV) mortality increased in people with disability [17–21].

Being one of most widely used low extremity mobility tests, the timed up and go (TUG) test examines the physical performance status of activities in everyday life. The test can be easily performed in clinical settings, and used to examine static balance, dynamic balance, strength of lower extremities and gait speed. TUG has been reported to predict falls, fractures, hospitalisation by fractures [22], low quality of life [23], decreased social participation [24] and difficulty of activity of daily living (ADL) [25].

Low extremity mobility test was reported to predict the future incidence of disability. Previous studies have shown that low extremity mobility tests, such as gait speed or TUG, predicted the incidence of disability measured by ADL, or instrumental ADL (IADL) [26–31]. Different Korean and Japanese studies have reported that abnormal TUG was associated with future receipt of long-term care service (LTCS) [32, 33]. However, previous studies have used either subjective measure disability such as ADL or IADL or indirect measure such as LTCS use.

The purpose of this study is to examine the association between TUG and disability incidence longitudinally, by using National Screening Programme for Transitional Ages (NSPTA) and KNDR, which are national representative data regarding disability which is diagnosed and proved by physician including physical impairment, brain impairment, visual impairment, hearing impairment, linguistic impairment, mental impairment and others (i.e., developmental disability, renal function impairment, heart function impairment, respiratory function impairment, liver function impairment, facial deformity, intestinal and urinary tract function impairment and epilepsy).

## Materials and methods

### Study setting

The Korean National Health Service is a public health insurance system (i.e., Korean National Health Insurance Service, KNHIS), which provides universal health coverage to almost all Koreans except for Medicaid beneficiaries, who account for less than 3% of the population. The KNHIS provides National Screening Programme (NSP), which is a biennial health screening programme, and the NSPTA was added to NSP in 2007. The objectives of the NSPTA were to tailor the programme according to the age and sex of each subject and strengthen post-examination counselling. In NSPTA, specific examinations were performed to participants according to their age and sex, which means tailored examinations. The program also provides on-demand additional counselling of physicians when participants have poor lifestyles such as inactivity, tobacco smoking, or at-risk alcohol drinking. KNHIS sends a letter to participants to invite to this screening program. And participants enter the screening program, when they visit a screening facility, which is usually nearby hospital. In the program, participants answer a questionnaire about their health-related lifestyle and past medical history, and received tailored examinations including body measurements (height, weight, waist circumference, and blood pressure), blood sampling tests (blood sugar, cholesterol, and liver/renal tests), and radiological examinations (dual X-ray absorptiometry and chest radiograph). After finishing the examination, each participants receives formal report on the examination results which is prepared by screening facility. As a part of this programme, only participants aged 66 years undergo the TUG and unipedal stance tests to assess mobility. For this reason, all participants of this study were 66 years old. KNHIS created the National Health Information Database (NHID), which includes data on healthcare utilisation, health screening results in NSTPA, socio-demographic variables and mortality rates for over 50 million people in Korea [34]. The details of the NSPTA have been described elsewhere in research works [35].

### Data sources and study population

**Fig 1:**
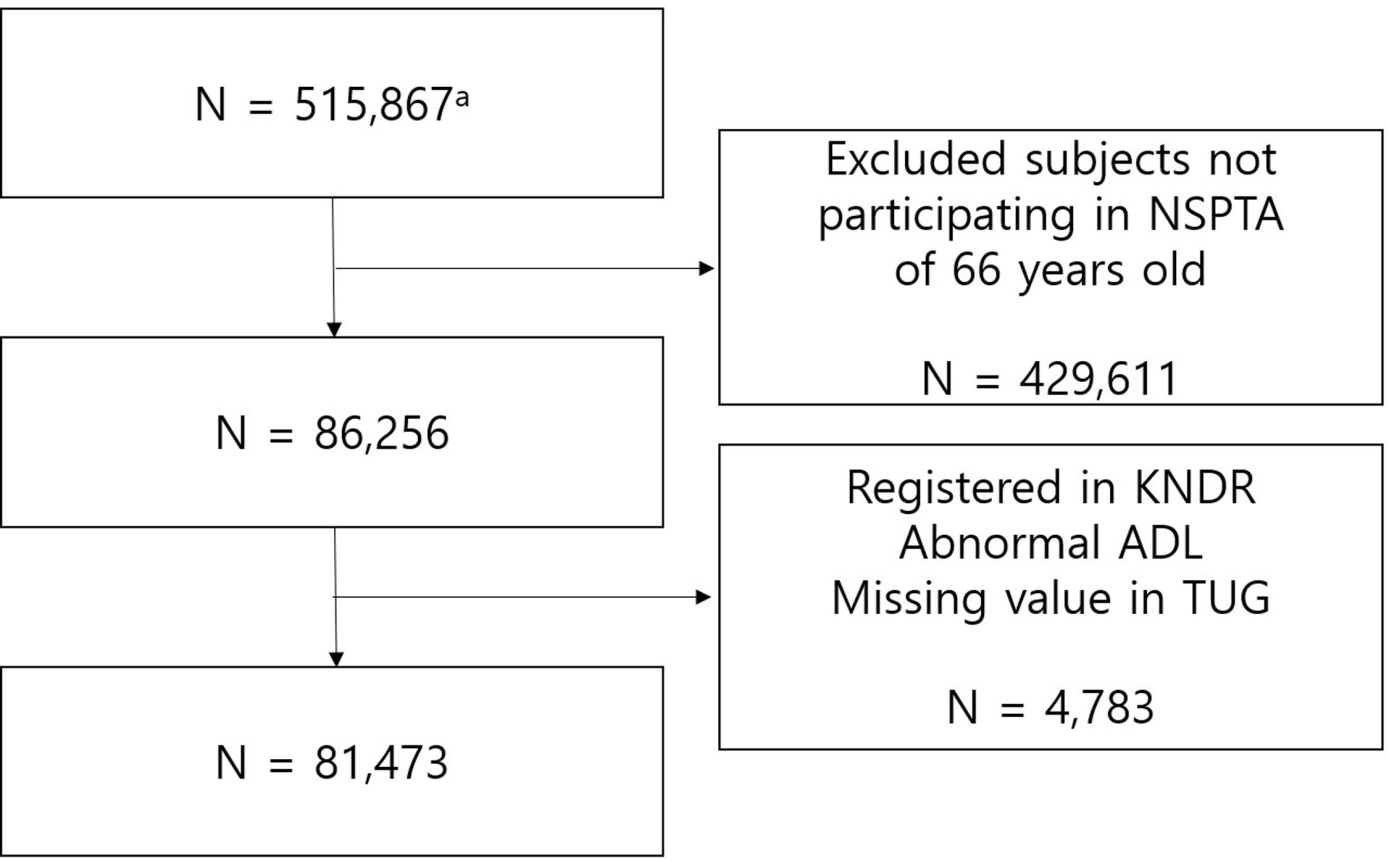
Flow of subject selection. Number of subjects in original the National Health Insurance Service–National Health Screening Cohort (2002–2015). * Abbreviations: NSPTA: National Screening Programme in Transitional Ages, KNDR: Korean National Disability Registry, ADL: Activity of Daily Living.

We used the National Health Insurance Service–National Health Screening Cohort (NHIS–HEALS) database of NHID, in which 515,867 participants were included. This represents 10% of a randomly selected people from total Korean participants aged 40–79 years who participated in NSP at least once between 2002 and 2003. Among them, we included participants who underwent NSPTA when they were 66 years old during the time between 2007 and 2015. We excluded participants who were with a disability at the time of NSPTA examination. After excluding participants who already had registered as a person with disability in the KNDR, with missing or misclassification, or with abnormality in ADL, we included 81,473 participants for analysis in our study (Fig 1).

The database included demographic data of the participants (i.e., age, sex, residential area and income status), information of disability, survey data regarding medical history and health behaviours, and screening results, including height, weight, abdominal circumference, physical function test (i.e., TUG test and unipedal stance test) and laboratory tests. In addition, we could secure information of medical facilities utilisation and death.

The Institutional Review Board of Seoul National University Hospital approved the protocol of this study (IRB No. E-1703-020-836), and the requirement for informed consent was waived because the KNHIS database was constructed after ensuring anonymisation according to strict confidentiality guidelines.

### Variables

#### Independent variable

*Timed up and go test*. The TUG was performed on the day of physical examination during the NSPTA at each subject’s community hospital. It was conducted as per the guidelines provided in the NSPTA manual. Participants were required to sit on a chair, stand and walk a 3-m course at a comfortable speed, walk back to the chair and sit again, while wearing regular footwear and/or using walking aids. The time from standing up to sitting down again was measured, and a time period greater than 10 s was categorised as abnormal.

#### Outcome variables

In this study, we used the incidence of disability as an outcome variable. The NHIS–HEALS dataset includes the information of disability of NSPTA participants such as severity and type of disabilities and registration month, which is merged from KNDR. To register to KNDR, the persons have to be diagnosed with any disability by their physician, and acquire document written by their physician that proves their disability status. The document is submitted to and audited by the Center for Disability Registration Audit (CDRA). If the CDRA approves the document, then the person is registered to KNDR. In this reason, the registration of KNDR is an objective measure of disability status compared with ADL or IADL, which is subjective reporting of their performance status. According to the statistics of National Rehabilitation Center, about 2.4 million people in Korea were registered to KNDR in 2016, which is estimated to over 94% of people with disability in Korea [5]. This observation suggests that nearly all people with disability are registered in KNDR. The severity of disability was divided into six grades in KNDR from one to six: the grade one of disability is the severest, whereas grade six is the least severe. In the NHIS–HEALS dataset, grades one and two are categorised as ‘severe disability’, whereas grade three to six as ‘mild disability.’ If participants did not have any grade of disability, then they were categorised as ‘not disabled’, which means they are persons without disability. Types of disability were divided into eight categories: physical impairment, brain impairment, visual impairment, hearing impairment, linguistic impairment, mental impairment and others (i.e., developmental disability, renal function impairment, heart function impairment, respiratory function impairment, liver function impairment, facial deformity, intestinal and urinary tract function impairment and epilepsy).

We assumed that there is no right censoring other than death. Because all persons are supposed to be beneficiaries of the National Health Insurance in Korea, dropout other than death is virtually impossible. The follow-up time was calculated as the time from the date of the NSPTA examination to first day of registration month for disability, date of death of the individual, or the end of 2015, whichever comes first.

### Potential confounders

We collected the data of chronic diseases, such as hypertension, diabetes mellitus and dyslipidemia, by using the questionnaire and laboratory results of NSPTA. Because of limitation of NHID claim data as a reliable source of case definition, we did not collect confounding variables which are not available in screening results. If participants answered that they took medications for hypertension, diabetes mellitus or dyslipidemia, then they were considered to have the diseases. Participants with systolic blood pressure ≥ 140 mmHg or diastolic blood pressure ≥ 90 mmHg were considered to have hypertension. Participants with serum fasting glucose level ≥ 7.0 mmol/L were considered to have diabetes mellitus. Moreover, participants with serum total cholesterol level ≥ 13.3 mmol/L were considered to have dyslipidemia.

We calculated body mass index (BMI) the weight divided by height squared (kg/m^2^). We used Asian-specific criteria for BMI to define normal BMI, in which normal BMI is defined between 18.5 and 23 kg/m^2^, underweight as less than 18.5 kg/m^2^, and obese as BMI ≥ 25 kg/m^2^.

We measured cognition by using the Korean Dementia Screening Questionnaire-Cognition (KDSQ-C), which is included in the NSPTA questionnaire. The KDSQ-C is a self-administered, validated questionnaire [36], comprising 15 items, each rated on a three-point Likert scale (0, 1, or 2, with a higher score considered worse). Cognitive impairment was defined as a composite score ≥6.

We measured depressive mood by using three questions extracted from Korean Version of Geriatric Depression Scale (GDS), which is validated elsewhere [37]. The questions were: ‘Do you feel that your activity or desire decreased recently?’, ‘Do you feel that you are useless for now?’ and ‘Do you feel that you are hopeless in current situation?’ Although GDS is not diagnostic but suggests necessity of a full assessment of depression, if there was any ‘Yes’ for these questions, then the participants were categorised as depressed.

The NSPTA questionnaire included six items about ADL, which were extracted from the Korean versions of the ADL (K-ADL) and Instrumental ADL (K-IADL) questionnaires [18]. The four items extracted from the K-ADL were: ‘Do you bathe by yourself without help?’, ‘Do you dress by yourself without help?’, ‘Do you eat by yourself without help if a meal is prepared?’ and ‘Do you go to the toilet by yourself without help?’ The two items extracted from the K-IADL were ‘Do you prepare your meal by yourself without help?’ and ‘Do you go outside by yourself to places within walking distance?’ ADL was categorised as abnormal if the answer to one or more of these questions was ‘No.’

The database was reviewed for International Classification of Diseases 10th Revision codes for the diagnosis of CV disease. CV disease was defined as a diagnosis of ischaemic heart diseases (I20-25) or cerebrovascular disease (I60-69).

### Statistical analysis

In the baseline characteristics, continuous variables were reported as mean ± SD, whereas categorical variables were described as frequencies and percentages. The incidence rates of disability were described as per 1,000 person-years (PYs).

We used Cox proportional hazard models to evaluate the association between TUG results and future disability incidence. In survival analyses, we built four different models. In addition to Crude model, Model 1 was adjusted for sex. In Model 2, depressive mood and cognitive impairment were added to Model 1. Also in Model 3, hypertension, diabetes mellitus, dyslipidemia and BMI were added to Model 2. We calculated the hazard ratio and 95% confidence interval (CI) for each model.

We conducted subgroup analyses. First, we performed survival analysis according to the sex group. Then, we moved to risk factors including depressive mood and cognitive impairment, to chronic diseases including obesity, hypertension, diabetes mellitus, dyslipidemia and to CV disease.

We performed statistical analyses by using STATA software (Version 15.1; STATA. Corp, College Station, TX). P < 0.05 was considered statistically significant for this study.

## Results

### Basal characteristics of participants

The mean follow-up duration was 4.1 ± 2.6 years (maximum, 8.9 years). At the end of 2015, 86 participants (0.1%) were newly registered in KNDR. In the follow-up of 334,200.9 PYs, the incidence rate of disability was 0.208 per 1,000 PY.

Among the 81,473 participants included in analysis, 23,664 (29.0%) were participants with abnormal TUG results (i.e., participants taking more than 10 s to complete the test), and the mean of TUG results was 8.44 ± 3.08 s. About 50% of the participants were women, and women had more abnormal TUG results than men. Over two-thirds of the participants were normal in their baseline body weight, whereas one-third of them were overweight. Only 2% of the participants were underweight. In total, 20% of participants showed depressive mood, and 15% of them had cognitive impairment. In total, 70% of the participants answered that they had hypertension, 26% of them had diabetes mellitus and 28% of the participants had dyslipidemia. Additionally, 649 (0.8%) participants had CV disease.

Eventually, 86 (0.1%) participants registered to KNDR, and 50 (0.1%) of them were normal in TUG, whereas 36 were abnormal (Table 1).

**Table 1:** Baseline characteristics of participants.

	Total	Timed up and go	
		Normal	Abnormal
	N (%)	N (%)	N (%)
Sex (women) (%)	41,063 (50.4)	28,947 (48.8)	12,122 (54.8)
Timed up and go test (sec)	8.44 ± 3.08	7.20 ± 1.49	11.76 ± 3.73
BMI (kg/m^2^)			
Underweight (<18.5)	1,678 (2.1)	1,211 (2.0)	467 (2.1)
Normal weight (18.5–25)	49,652 (60.9)	36,546 (61.6)	13,106 (59.2)
Overweight (≥25)	32,134 (37.0)	21,578 (36.4)	8,565 (38.7)
Depressive mood	16,458 (20.3)	11,342 (19.2)	5,116 (23.2)
Cognitive impairment	12,247 (15.1)	8,488 (14.4)	3,759 (17.1)
Hypertension	58,695 (71.7)	42,023 (70.8)	16,372 (74.0)
Diabetes mellitus	21,065 (25.9)	14,638 (24.7)	6,427 (29.0)
Dyslipidemia	23,026 (28.3)	16,149 (27.2)	6,877 (31.1)
Cardiovascular disease	649 (0.8)	484 (0.8)	165 (0.8)
Participants with disability	86 (0.1)	50 (0.1)	36 (0.2)

*Abbreviation: BMI, body mass index.

### Participants by type of disability

Among the normal TUG participants, 15 (30.0%) had physical impairment. And visual impairment and linguistic impairment were following. On the contrary, only one participant had physical impairment in the abnormal TUG group, whereas brain impairment, hearing impairment and mental impairment were the most frequent types of disabilities (Table 2).

**Table 2:** Proportion of participants by types of disability.

Type of disability	Normal N (%)	Abnormal N (%)
Physical impairment	15 (30.0)	1 (2.8)
Brain impairment	2 (4.0)	6 (16.7)
Visual impairment	6 (12.0)	2 (5.6)
Hearing impairment	4 (8.0)	5 (13.9)
Linguistic impairment	5 (10.0)	2 (5.6)
Mental retardation	0 (0)	3 (8.3)
Mental impairment	1 (2.0)	4 (11.1)
Others	17 (34.0)	(36.1)

*Physical impairment: amputation, motor disturbance, joint disability, deformity of limbs, spinal cord injury; Brain impairment: brain disability caused by stroke, brain damage, brain palsy; visual impairment: loss of visual power, visual field defect; hearing impairment: hearing disability, disability of sense of equilibrium; linguistic impairment: mogilalia, dysphonia; mental retardation: intelligence quotient < 70; mental impairment: mental disease with limitation of daily life such as depression.

### Timed up and go test and incidence of disability

In the normal TUG group, the disability incidence rate was 0.215 per 1,000 PY, whereas it was 0.354 in the abnormal TUG group. Compared with the normal TUG group, the abnormal TUG group showed an increase in disability in all models. In Model 3, the fully adjusted model, an increase of 60% was observed (adjusted hazard ratio [aHR]: 1.600, 95% confidence interval [CI] 1.036–2.472) (Table 3).

**Table 3:** Association between timed up and go test and disability incidence.

	Disability	Number	Disability	Duration (PY)	Incidence rate	Crude	Model 1	Model 2	Model 3
						HR (95%CI)	aHR (95%CI)	aHR (95%CI)	aHR (95%CI)
Total	All	81,473	86	334,200.9	0.208				
	Not disabled	62,910	50	232,551.6	0.215	Ref	Ref	Ref	Ref
	Disabled	23,664	36	101,649.3	0.354	1.665 (1.084–2.559)	1.742 (1.133–2.679)	1.640 (1.062–2.531)	1.600 (1.036–2.472)
Sex									
Men	All	40,410	57	161,655.8	0.353				
	Not disabled	32,493	34	116,889.6	0.291	Ref	N/A	Ref	Ref
	Disabled	10,825	23	44,766.2	0.514	1.738 (1.022–2.955)		1.618 (0.944–2.772)	1.578 (0.920–2.705)
Women	All	41,063	29	172,545.2	0.168				
	Not disabled	30,417	16	115,662.0	0.138	Ref	N/A	Ref	Ref
	Disabled	12,839	13	56,883.1	0.229	1.747 (0.839–3.636)		1.659 (0.796–3.454)	1.623 (0.778–3.384)

*Cox proportional hazard was applied.

Model 1: sex

Model 2: Model 1 +depressive mood, cognitive impairment

Model 3: Model 2 + BMI, hypertension, diabetes mellitus, dyslipidemia

**Abbreviation: PY, person-year; HR, hazard ratio; aHR, adjusted hazard ratio; CI, confidence interval. N/A: not applicable.

### Subgroup analysis

**Fig 2:**
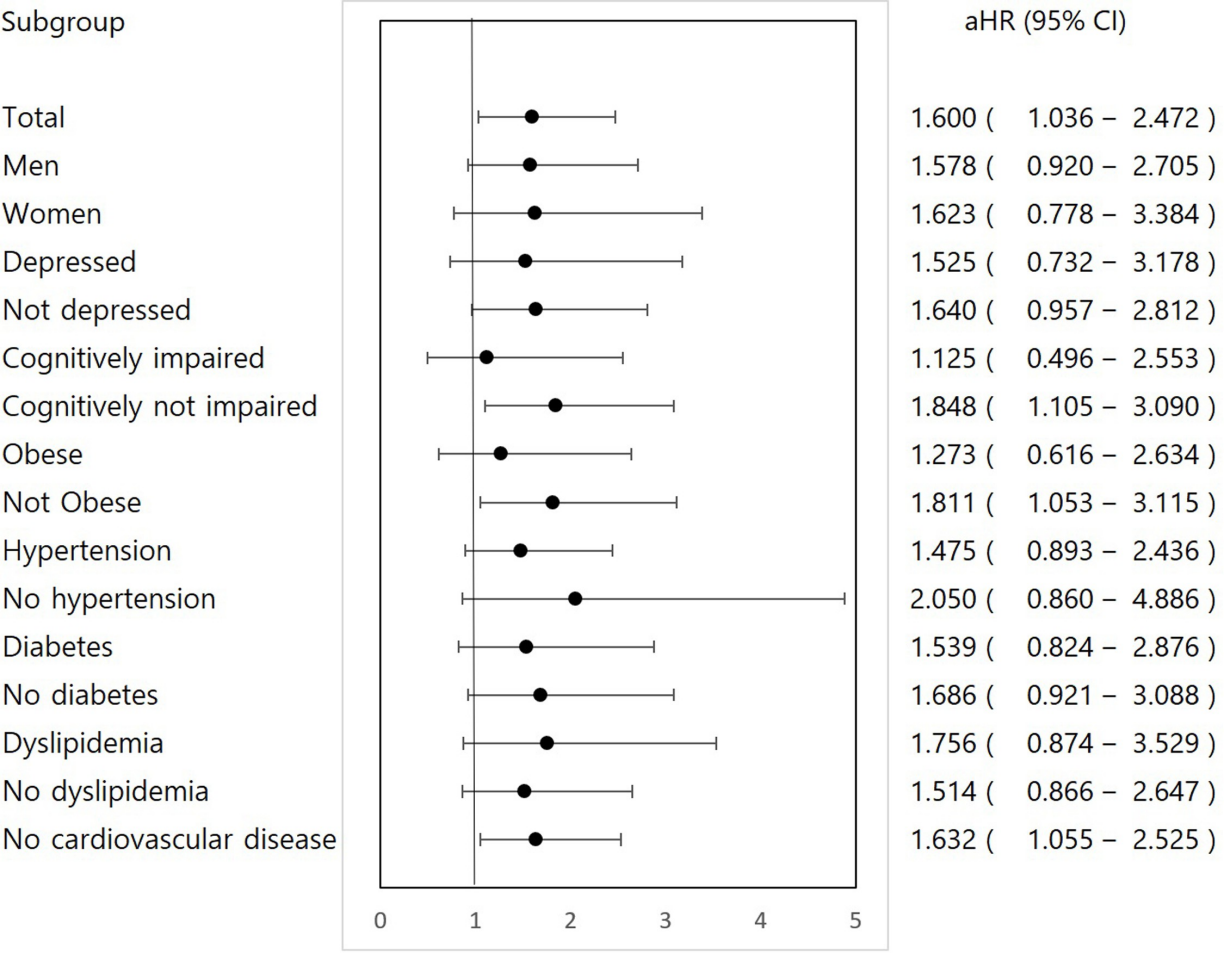
Forest plots showing subgroup analyses of the association of timed up and go test results with disability. *Abbreviations: aHR, adjusted hazard ratio; CI, confidence interval.

In the subgroup analysis, the incidence of disability increased 1.85 times in the cognitively unimpaired participants (aHR: 1.848, 95%CI: 1.105–3.090). Also, non-obese participants showed a higher disability incidence of 81% in the abnormal TUG group than in the normal TUG group (aHR: 1.811, 95%CI: 1.053–3.115). When participants did not have CV diseases, their disability incidence was 63% higher in abnormal participants (aHR: 1.632, 95%CI: 1.055–2.525). On the contrary, the incidence was not different in participants with cognitively impairment or obesity. Disability incidence was not different regardless of sex, depressive mood and chronic diseases (i.e., hypertension, diabetes mellitus and dyslipidemia). There was only one participant who registered to KNDR in the CV disease group; therefore, we could not calculate aHR for the group (Table 3 and Fig 2).

## Discussion

To our knowledge, this is the first large general population study that evaluated the association of TUG test with disability incidence in Asian people by using a nationwide representative claim database. We used the objective measures of disability status, registration in KNDR rather than ADL/IADL or LTCS use. Overall, disability incidence increased by 60% in participants with abnormal TUG results. The increase was more evident for participants with normal cognition, without obesity or CV disease. This study added evidence that TUG as a marker of physical frailty, predicts the future disability which is strictly and objectively defined.

Previous studies reported that low extremity mobility test was associated with the future incidence of disability. However, most of the studies used gait speed as mobility test and ADL/IADL use as the marker of disability [26–31], and there are only a small number of studies using TUG as a mobility measure. The only study using TUG as the mobility test to examine the association with ADL/IADL, was an Irish study which showed that TUG predicted the incidence of disability measured by ADL and IADL after two years [26]. Although ADL/IADL are widely used measures of disability, they are subjective measures of self-reporting inability for specific daily tasks. In contrast, registration in KNDR is an objective measure of disability, which is diagnosed by physician and confirmed by CDRA. As far as we know, this is the first longitudinal study using the objective measure of disability to examine the association between TUG and disability incidence.

Lee et al. evaluated the association between TUG and LTCS use in Korea [32]. After 5.7 years of follow-up, they found that abnormal TUG was associated with 65% increase of LTCS use, which is similar to findings of this study. Although LTCS use is an excellent marker, which reflects functional decline in older adults, it is not a direct marker of incidence of disability because it is possible that people with disability do not use LTCS or delay the use. On the contrary, registration in KNDR is a direct marker of disability incidence because most people with disability in Korea are registered to KNDR.

In this study, there were only 86 cases of people with disability in the study population. This low incidence prevented us from a detailed analysis such as subgroup analysis according to the type of disability. There are two possible reasons for low disability incidence. First, the participants of this study were relatively old for a newly developed disability. In Korea, people with some types of disability die before reaching 60 years. For example, the mean age of death for people with autism, mental impairment, liver impairment, or epilepsy was less than 60 years [5]. This observation means that some type of disability was developed at a much younger age in this study population. Second, the follow-up period, which was 4.1 years, was relatively short for this young–old population. This prevents detecting delayed the incidence of disability.

While disability incidence was significantly associated with TUG results in the entire study population, this association was significant only in several subgroups, such as people with normal cognition, without obesity or CV disease, thereby suggesting that physiologic changes associated with TUG abnormality are not a strong risk of disability than these conventional risk factors. Therefore, TUG results would not affect disability incidence rates in participants with these risk factors. However, the 60–80% higher rate of disability incidence in participants with abnormal TUG results lacking strong risk factors such as cognitive impairment, obesity, or CV disease suggests that TUG results may help in identifying participants who are a higher risk of having disability without any strong risk factors. There is possibility that limitation of confounding factors, small number of cases and short duration of follow up prevented finding other important modifying factors. Therefore, there is a need for future study with larger case number, longer follow up and more potential confounders considered.

There are several limitations in this study. There is a limitation of database, which prevented the adjustment for risk factors such as household income, education, dietary intake. Moreover, disability incidence was lower than expected, it was impossible to evaluate the association with TUG according to the type of disability. This is partly because of strict criteria to be registered to KNDR, which is predetermined by law. And it is possible that valuable information was missed due to restrictive definition of disability, and it undermines the credibility of representativeness of the disabled population in this study. Furthermore, the strictness of KNDR registration could make difference in definition of disability from definition largely used. Additionally, because NHID is a medical claim database, it is possible that there are misclassifications of diagnoses of CV disease. However, every claim in this dataset was audited by the Korean Health Insurance Review and Assessment before payment, thereby making the misclassification of diagnosis improbable.

In conclusion, there was an increase in disability incidence in participants with abnormal TUG results. The increase was more evident for participants with normal cognition, without obesity or CV disease. Caution should be exercised to interpret these results because of limitation of dataset which is lack of some relevant covariates.
