# Correction: Association between timed up and go test and future incidence of disability: A nationwide representative longitudinal study in Korea

**DOI:** 10.1371/journal.pone.0314934

**Published:** 2024-12-02

**Authors:** Ki Young Son, Dong Wook Shin, Ji Eun Lee, Sang Hyuck Kim, Jae Moon Yun, Belong Cho

There is an error in affiliation 1 for author Ki Young Son. The correct affiliation 1 is: Department of Family Medicine, Asan Medical Center, Seoul, Korea, University of Ulsan College of Medicine, Seoul, Korea
